# LncRNA MIR100HG affects the proliferation and metastasis of lung cancer cells through mediating the microRNA‐5590‐3p/DCBLD2 axis

**DOI:** 10.1002/iid3.1223

**Published:** 2024-04-11

**Authors:** Shengping Min, Linxiang Zhang, Li Zhang, Fangfang Liu, Miao Liu

**Affiliations:** ^1^ Department of Microbiology and Parasitology, School of Basic Medical Sciences Anhui Medical University Hefei Anhui China; ^2^ Department of Pulmonary and Critical Care Medicine, First Affiliated Hospital, Bengbu Medical College Anhui Province Key Laboratory of Clinical and Preclinical Research in Respiratory Disease Bengbu Anhui China

**Keywords:** DCBLD2, lung cancer, lncRNA MIR100HG, metastasis, miR‐5590‐3p, proliferation

## Abstract

**Objective:**

The aim of this paper is to investigate the effect of long noncoding RNA (lncRNA) MIR100HG on the proliferation and metastasis of lung cancer cells by mediating the microRNA (miR)−5590‐3p/DCBLD2 axis.

**Methods:**

RNA levels of MIR100HG, miR‐5590‐3p, and DCBLD2 in lung cancer tissues and cells were detected by quantitative reverse‐transcription polymerase chain reaction, and protein level was assessed by Western blot. Effects of MIR100HG or miR‐5590‐3p on proliferation, migration, and invasion of lung cancer cells were detected by Cell Counting Kit‐8, colony formation, and Transwell assays. Luciferase reporter assay and RNA‐immunoprecipitation assay confirmed the target relationship between miR‐5590‐3p and MIR100HG or DCBLD2.

**Results:**

MIR100HG and DCBLD2 were highly expressed, while miR‐5590‐3p was lowly expressed in lung cancer tissues and cells. Silencing MIR100HG or upregulating miR‐5590‐3p impeded lung cancer cell proliferation, migration, and invasion. MIR100HG could up‐regulate DCBLD2 by sponging miR‐5590‐3p. Downregulation of miR‐5590‐3p partly overturned the suppressive effect of silencing MIR100HG on lung cancer cell proliferation and metastasis, and overexpression of DCBLD2 also reversed the effect of overexpression of miR‐5590‐3p on lung cancer cell proliferation and metastasis.

**Conclusion:**

LncRNA MIR100HG promotes lung cancer progression by targeting and negatively regulating DCBLD2 through binding with miR‐5590‐3p.

## Graphical abstract

The novelty of this study is that MIR100HG promotes lung cancer progression by modulating the miR‐5590‐3p/DCBLD2 axis. Besides, downregulation of miR‐5590‐3p reversed the suppressive effect of silencing MIR100HG on lung cancer cell proliferation and metastasis, and overexpression of DCBLD2 also reversed the effect of overexpression of miR‐5590‐3p on lung cancer cell proliferation and metastasis.

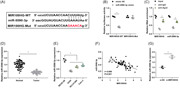

## 1 INTRODUCTION

Lung cancer is a frequently diagnosed tumor and a chief reason for cancer‐related deaths around the world, ad there are an estimated 2 million suffers and 1.76 million deaths annually.^1^ Lung cancer involves diverse treatment modalities, consisting of surgery, interventional radiology, radiotherapy, palliative care, as well as systemic therapies (immunotherapy, chemotherapy, and targeted agents).^2^ Centralized clinical management of postsurgical lung cancer patients by nurses remains a priority, regardless of the technique used.^3^ Lung cancer tumors are allocated into two histological categories, consisting of non‐small‐cell lung carcinoma (NSCLC) and small‐cell lung carcinoma (SCLC).^4^ lung cancer's all‐round molecular characterization has expanded our recognition of the cellular origins and molecular pathways impacted in these subtypes.^5^ Nevertheless, lung cancer remains a primary reason for cancer death even with great advances in our recognition of risk, immunologic control, development, as well as treatment options for this disease.^6^

Long noncoding RNA (lncRNA) has gained much attention due to the rapid development of whole genome and transcriptome sequencing technology.^7^ LncRNAs are involved in diverse biological processes (e.g., cell proliferation, survival, differentiation, metastasis, and apoptosis) but are also implicated in tumorigenesis and metastasis in multiple cancer types.^8^ In lung cancer‐related research, numerous differentially expressed lncRNAs have been discovered, and several of them are regarded as oncogenic lncRNAs, while other lncRNAs are called tumor‐suppressive lncRNAs.^9^ MIR100HG plays a tumor‐driving or tumor‐suppressive role in various cancers, which participates in diverse tumor cell biology processes together with cancer‐related pathways.^10^ MIR100HG might shed lights on new targets for individualized immunotherapy for bladder cancer patients,^11^ and MIR100HG participates in the suppression of the immune escape of gastric cancer cells.^12^ Nevertheless, the specific modulatory function of MIR100HG remains unknown in lung cancer. The modulatory mechanisms of lncRNAs in diseases comprise competing endogenous RNAs (ceRNAs), chromatin modification, and interference with mRNA expression.^13^ As previously described, MIR100HG is validated to target microRNA (miR)‐5590‐3p, and its expression has a negative correlation with miR‐5590‐3p.^14^ Circulating miRNAs are revealed to be dysregulated and are bound up with lung cancer patients' clinicopathological parameters and overall survival.^15^ miRNAs are implicated in both physiological and pathological processes, such as cellular differentiation, proliferation, apoptosis, autophagy, and ferroptosis.^16^, ^17^, ^18^ miR‐5590‐3p has been detected to be downregulated in many types of cancers, including gastric cancer and breast cancer.^19^, ^20^ miR‐5590‐3p has been revealed to participate in the promotion of diffuse large B cell lymphoma progression and immune evasion.^21^ However, its relationship with lung cancer remains to be unsettled. Discoidin, CUB, and LCCL domain containing 2 (DCBLD2), also called ESDN or CLCP1, was first discovered in human coronary arterial cells via a signal sequence trap approach. DCBLD2 is mapped to human chromosome 3q12.1;3,^22^ which has been verified to be an inflammation‐related gene.^23^ DCBLD2 has been demonstrating to mediate biological functions of various tumors, including hypopharyngeal squamous cell carcinoma and myxofibrosarcoma.^24^, ^25^ Besides, DCBLD2 has been revealed to participate in lung cancer development.^26^ Herein, we aimed to investigate the effect of MIR100HG on the proliferation and metastasis of lung cancer cells by mediating the miR‐5590‐3p/DCBLD2 axis.

## 2 MATERIALS AND METHODS

### 2.1 Ethical approval

All patients have signed a written informed consent form, and in addition, this study has been approved by the ethics committee of Bengbu Medical College([2023] 389).

### 2.2 General data

Lung cancer tissue specimens and paired normal paracancerous tissues (more than 5 cm from cancerous tissue) were collected from lung cancer patients who underwent resection surgery at the First Affiliated Hospital of Bengbu Medical College, and all samples were collected and immediately stored at −80°C for further experiments. None patients received preoperative chemoradiotherapy. The details of all patients are shown in Table 1.

**Table 1:** The relationship between MIR100HG expression levels and the clinicopathological characteristics of lung cancer patients.

General data	Case	LncRNA MIR100HG expression levels		*p*
		High expression group (*n* = 36)	Low expression group (*n* = 36)	
Age (years)				.479
≤60	37	20	17	
>60	35	16	19	
Gender				.624
Male	46	24	22	
Female	26	12	14	
Tumor diameter (cm)				.155
≤3	32	19	13	
>3	40	17	23	
TNM stage				<.001
I + II	44	14	30	
III + IV	28	22	6	
Lymph node metastasis				.017
No	30	10	20	
Yes	42	26	16	

### 2.3 Cell culture and treatment

Human lung cancer cell lines NCI‐H1299, A549, and CALU‐3 and normal human lung epithelial cell line (BEAS‐2B) were obtained from the Cell Bank of the Chinese Academy of Sciences. All lung cancer cells were cultured in RPMI‐1640 medium (Hyclone), and BEAS‐2B cells were cultured in Dulbecco's modified Eagle's medium (HyClone) and supplemented with 10% fetal bovine serum (FBS), 100 U/mL penicillin, and 100 μg/mL streptomycin (GIBCO). All cells were placed at the condition of 37°C and 5% CO_2_. All the above cell lines were verified for cell line contamination and showed no contamination.

Small interfering RNA (siRNA) against MIR100HG (si‐MIR100HG) (F‐5′‐ CCGGTTCCTCTGTTTGTACTTAAATCTCGAGATTTAAGTACAAACAGAGGAATTTTTG‐3′, R‐5′‐AATTCAAAAATTCCTCTGTTTGTACTTAAATCTCGAGATTTAAGTACAAACAGAGGAA‐3′) and matched negative control (si‐NC), or overexpression of DCBLD2 (oe‐DCBLD2) and its corresponding control (oe‐NC), or miR‐5590‐3p mimic and miR‐5590‐3p inhibitor with their corresponding controls (mimic NC, inhibitor NC) were synthesized in GenePharma (Shanghai, China), and all these various constructs were transfected into cells at logarithmic growth stage. Transfection was performed using Lipofectamine 3000 transfection reagent (Invitrogen) following the manufacturer's protocol. They were divided into the following groups: si‐NC group, si‐MIR100HG group, mimic NC group, miR‐5590‐3p mimic group, si‐MIR100HG + inhibitor NC group, si‐MIR100HG + miR‐5590‐3p inhibitor group, miR‐5590‐3p mimic + oe‐NC group, and miR‐5590‐3p mimic + oe‐DCBLD2 group.

### 2.4 Cell viability assay

Cell viability was tested using a cell counting kit‐8 (CCK‐8; DOJINDO) as per the manufacturer's instructions. Cells were seeded in 96‐well plates at a density of 4 × 10^3^ cells per well. After 24, 48, and 72 h of incubation, 10 μL of CCK‐8 reagent was supplemented to each well, and the optical density (OD) value of the samples was assessed at 450 nm using a spectrophotometer (Thermo Fisher).^14^

### 2.5 Colony formation assay

Transfected cells were seeded into six‐well plates (500 cells/well) and culture medium was replaced every 3–4 days. Fourteen days later, the culture medium was discarded, the cells were fixed in paraformaldehyde for 30 min and stained with 0.1% crystal violet (Solarbio) for 2 min, and the number of colonies was counted manually under a light microscope.^27^

### 2.6 Transwell assay

The cells were resuspended with serum‐free culture medium and adjusted for density, then inoculated into Transwell chambers coated with Matrigel. Cells at a density of 5 × 10^4^ cells/well (in 200 μL) were subjected to seeding in the upper transwell chambers where the membrane was coated with Matrigel (BD Biosciences) and the complete medium (500 μL) was supplemented to the bottom chambers. After that, the noninvaded cells in the upper chamber were wiped off with cotton swabs. On the contrary, the invaded cells on the membrane's bottom surface were fixed, dyed with crystal violet, and then observed using a microscope.^28^ The migration assay was performed without Matrigel coating, and the rest of the steps were the same as for invasion.

### 2.7 Quantitative real‐time polymerase chain reaction (RT‐qPCR)

Total RNAs were extracted from tissues and cells using Trizol reagent (Invitrogen). For the quantification of MIR100HG with DCBLD2, reverse‐transcription was performed using PrimeScript RT Master Mix kit (TaKaRa) as per the manufacturer's instructions, with GAPDH as the internal reference gene. For miR‐5590‐3p, miRNA First‐Stand cDNA Synthesis Kit (GeneCopoeia) was used with U6 as the internal reference gene. PCR primers were designed and synthesized by Sangon (The primer sequences were listed in Table 2). Real‐time PCR reactions were performed on Applied Biosystems 7500 Real‐time PCR Systems (Thermo Fisher Scientific). The 2^−ΔΔCt^ method was used to normalize the data, and the experiment was repeated three times.

**Table 2:** Primer sequences for genes in polymerase chain reaction assay.

Gene	Primer sequences (5′‐3′)
miR‐5590‐3p	Forward: 5′‐AATAAAGTTCATGTATGGCAA‐3′
	Reverse: Universal primer
U6	Forward: 5′‐GCTTCGGCAGCACATATACTAAAAT‐3′
	Reverse: 5′‐CGCTTCACGAATTTGCGTGTCAT‐3′
MIR100HG	Forward: 5′‐ACACAGACTTGTCTTTGGACA‐3′
	Reverse: 5′‐AAACCTGCTTCCATCTTGTTAG‐3′
DCBLD2	Forward: 5′‐GGAGCCCAGCAAGGTGATG‐3′
	Reverse: 5′‐GATGCGAACTCTCTCTCCCA‐3'
GAPDH	Forward: 5′‐GGTGTGAACCATGAGAAGTATGA‐3′
	Reverse: 5′‐GAGTCCTTCCACGATACCAAAG‐3′

### 2.8 Western blot

Proteins were extracted using radioimmunoprecipitation assay lysate (Beyotime). Protein concentrations were determined using the BCA Protein Concentration Assay Kit (Pierce). The samples were loaded according to the protein quantification results, and then treated with 10% sodium dodecyl sulfate‐polyacrylamide gel electrophoresis for 2 h. After that, the membrane was blocked with 5% skimmed milk powder at room temperature for 2 h, and incubated overnight at 4°C with primary antibody against DCBLD2 (1:100; Sigma‐Aldrich), followed by 1‐h incubation with the corresponding secondary antibody at room temperature and exposure for development, with GAPDH as the internal reference. The grayscale values were analyzed using the gel graphic analysis software ImageJ to calculate the relative protein expression.^29^

### 2.9 Dual luciferase assay

The sequences of MIR100HG and 3′‐untranslated region (3′‐UTR) of DCBLD2 containing predicted miR‐5590‐3p binding sites and their mutant sequences were sub‐cloned into the plasmid pmirGLO (Promega, USA) to generate MIR100HG wild‐type (MIR100HG‐WT), DCBLD2 wild‐type (DCBLD2‐WT), MIR100HG mutant (MIR100HG‐Mut), and DCBLD2 mutant (DCBLD2‐Mut) constructs. Lipofectamine 3000 was used to co‐transfect miR‐5590‐3p mimic and its control (mimic NC) and the above plasmids in cells, respectively. After transfection for 48 h, the relative luciferase activity was measured using a dual luciferase assay system (Promega).^30^

### 2.10 RNA immunoprecipitation (RIP)

RIP‐Assay Kit (Millipore) was utilized to perform RIP assay based on the manufacturer's instructions. The pre‐diluted anti‐IgG (Abcam) or anti‐Ago2 (Abcam) solution was supplemented to the cleaned beads and incubated for 2 h at low temperature. After 48 h of transfection, cells were fully lysed. The supernatant was cultivated with the beads conjugated with antibodies at 4°C overnight. Subsequently, the beads were harvested by centrifugation at low speeds and temperatures. RNA samples cleared from the beads were adopted for subsequent analysis of MIR100HG and miR‐5590‐3p.

### 2.11 Statistical analysis

SPSS 21.0 (SPSS, Inc) statistical software and GraphPad Prism 8.0 software were used to analyze the data. Measurement data were expressed as mean ± standard deviation. The *t*‐test was used for comparison between two groups, and one‐way analysis of variance followed by posthoc Turkey's test was used for comparison among multiple groups. The correlation analysis was performed using Pearson's correlation analysis. Enumeration data were expressed as rates or percentages, and the chi‐square test or Fisher's exact test was used for comparative analysis. Differences were considered statistically significant at *p* < .05.

## 3 RESULTS

### 3.1 MIR100HG is upregulated in lung cancer tissues and cells

**Figure 1:**
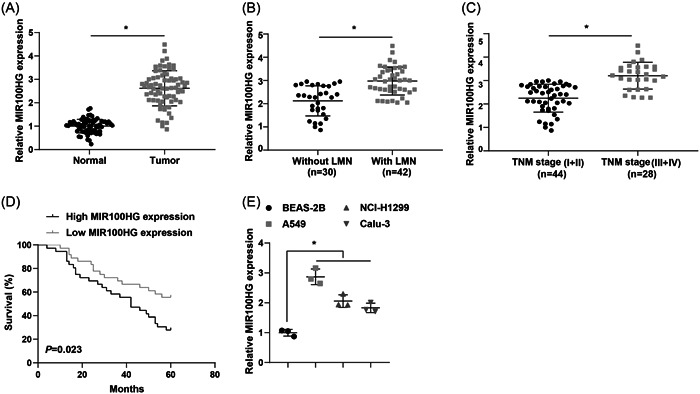
MIR100HG is upregulated in lung cancer tissues and cells. (A) RT‐qPCR was utilized to detect MIR100HG expression levels in lung cancer tissues versus corresponding paracancerous normal tissues (*n* = 72). (B) RT‐qPCR was implemented to assess MIR100HG expression levels in lung cancer tissues with or without lymph node metastasis. (C) RT‐qPCR was employed to determine MIR100HG expression levels in lung cancer tissues with different TNM stages. (D) The survival in lung cancer patients was analyzed by Kaplan–Meier analysis. (E) RT‐qPCR was used to evaluate MIR100HG expression levels in lung cancer cells. *p < .05. RT‐qPCR, Quantitative real‐time polymerase chain reaction.

It has been shown that MIR100HG is upregulated in TNBC and that silencing of MIR100HG reduces the proliferation of TNBC cells.^31^ However, no relevant studies focused on the role of MIR100HG in lung cancer. Based on this, we first examined the expression levels of MIR100HG in lung cancer tissues versus corresponding paracancerous normal tissues by RT‐qPCR, and the results disclosed that MIR100HG was significantly upregulated in lung cancer tissues compared with corresponding paracancerous normal tissues (Figure 1A). In addition, patients were divided into high and low‐expression groups according to the median value of MIR100HG expression levels. By analyzing the correlation between MIR100HG levels and different clinicopathological characteristics (age, gender, TNM stage, tumor size, and lymph node metastasis [LNM]), we found that high MIR100HG expression was associated with late TNM stage and the presence of LNM, but not with age, gender, and tumor size of lung cancer patients (Table 1 and Figure 1B,C). Further Kaplan‐Meier analysis showed that lung cancer patients with low MIR100HG expression had significantly higher survival rates than those with high MIR100HG expression (Figure 1D). Finally, lung cancer cell lines (A549, NCI‐H1299, and CALU‐3) and human normal bronchial epithelial cell lines (BEAS‐2B) were selected to explore the aberrant expression of MIR100HG at the cellular level, and the results reflected that MIR100HG was highly expressed in human lung cancer cells (A549, NCI‐H1299, and CALU‐3) versus BEAS‐2B cells (Figure 1E), and was most significantly upregulated in A549 cells in particular; therefore, A549 cells were selected for the follow‐up study.

### 3.2 Silencing MIR100HG inhibits lung cancer cell proliferation, migration, and invasion

**Figure 2:**
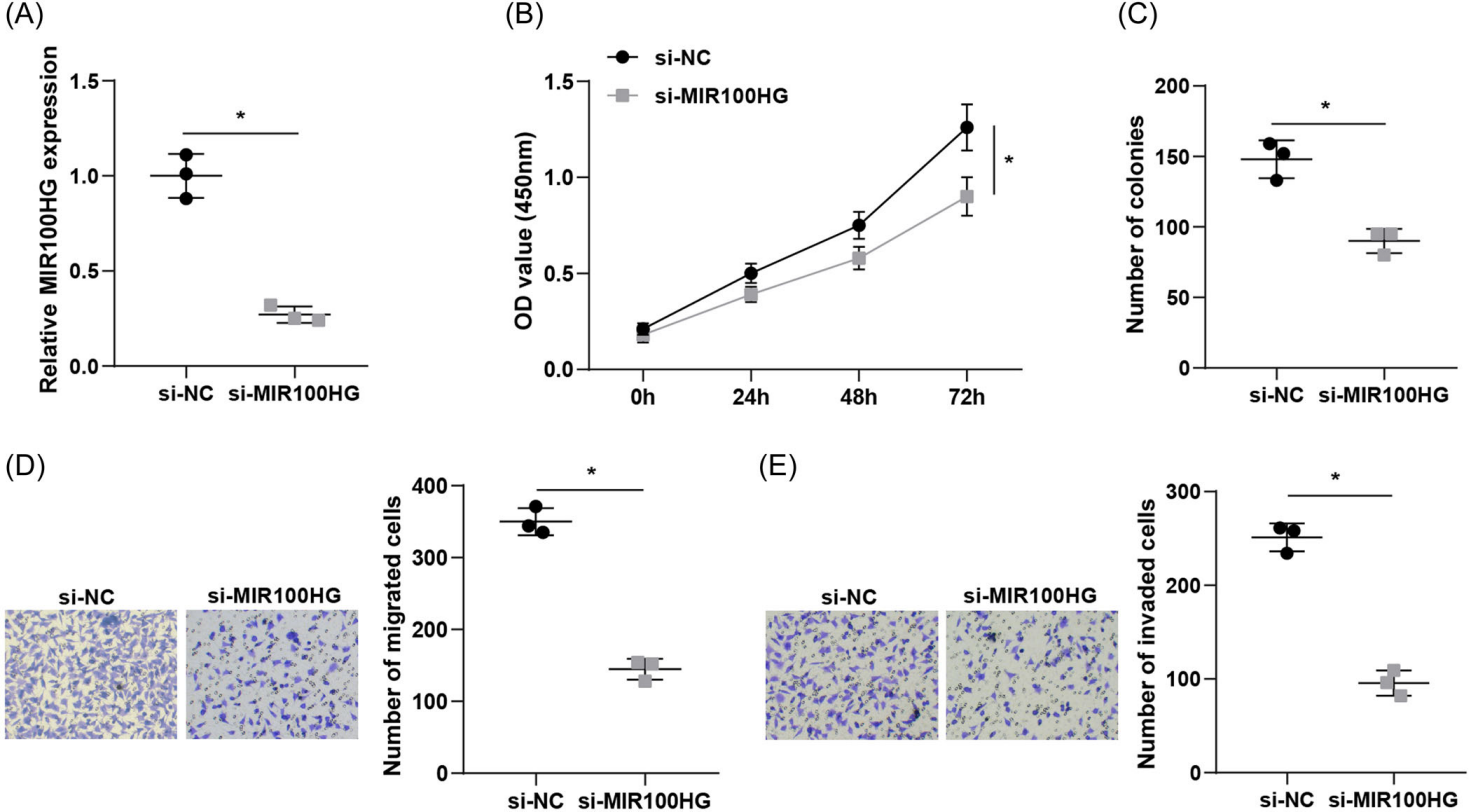
Silencing MIR100HG inhibits lung cancer cell proliferation, migration, and invasion. (A) RT‐qPCR was adopted to detect MIR100HG expression levels in cells. (B) CCK‐8 assay was employed to assess cell proliferation ability. (C) Colony formation ability of cells was examined by colony formation assay. (D–E) Transwell assay was implemented to evaluate cell migration and invasion ability. *p < .05. CCK‐8, Cell Counting Kit‐8; RT‐qPCR, Quantitative real‐time polymerase chain reaction.

To investigate the effect of MIR100HG on lung cancer cell progression, si‐NC and si‐MIR100HG were transfected into A549 cells, and RT‐qPCR demonstrated successful transfection (Figure 2A). CCK‐8 assay results showed that MIR100HG silencing impeded lung cancer cell proliferation (Figure 2B). Colony formation assay also revealed that MIR100HG silencing significantly suppressed the colony formation ability of lung cancer cells (Figure 2C). Transwell assay demonstrated that both invasive and migratory capabilities of A549 cells were significantly decreased after MIR100HG silencing (Figure 2D,E).

### 3.3 MIR100HG has a binding site to miR‐5590‐3p

**Figure 3:**
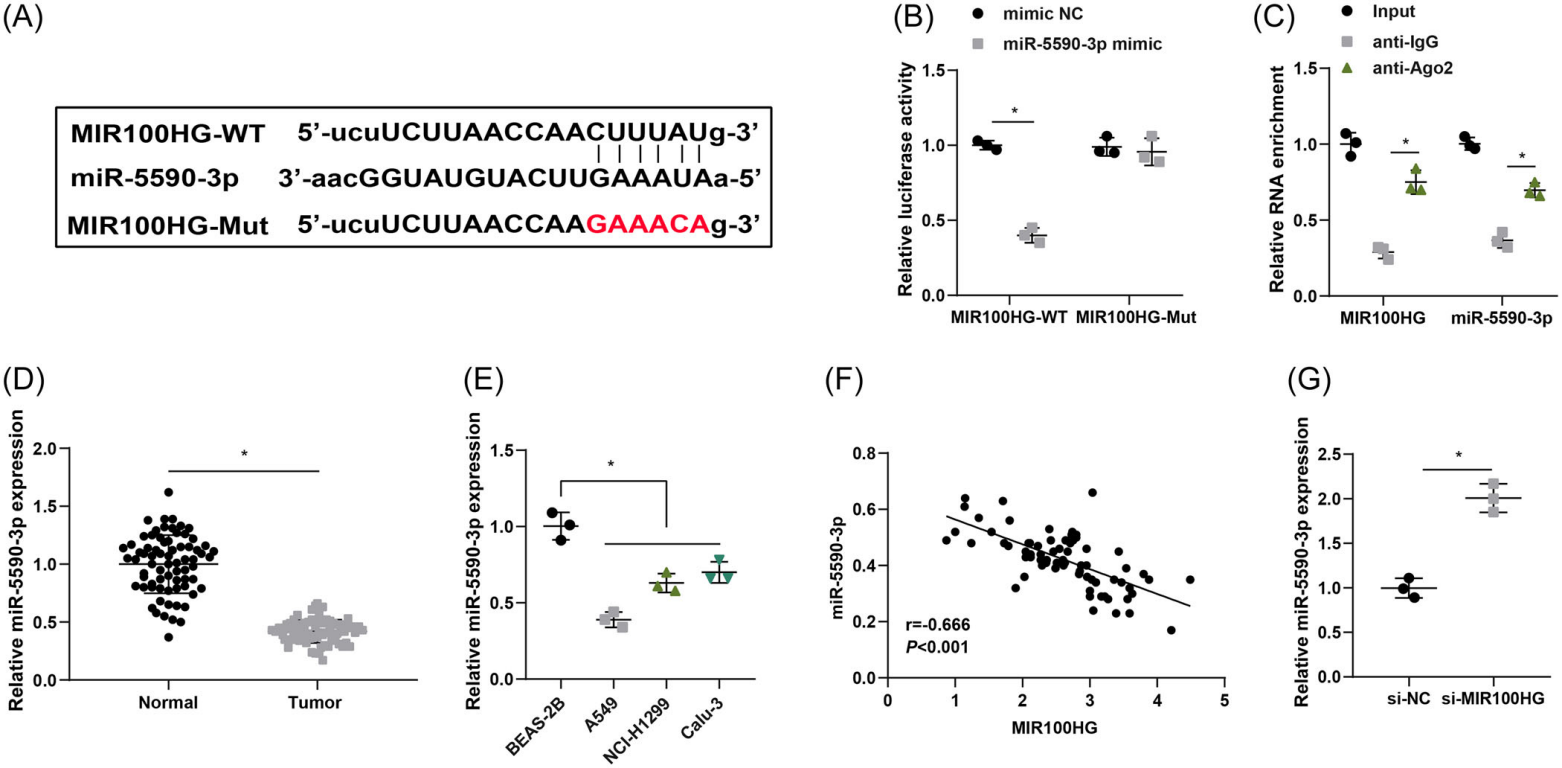
MIR100HG has a binding site to miR‐5590‐3p. (A) Binding relationship of MIR100HG and miR‐5590‐3p was predicted by the Starbase website. (B and C) Binding of MIR100HG and miR‐5590‐3p was detected by dual luciferase assay and RIP assay. (D) miR‐5590‐3p expression levels in lung cancer tissues and corresponding paracancerous normal tissues were determined by RT‐qPCR (*n* = 72). (E) miR‐5590‐3p expression levels in lung cancer cells were assessed by RT‐qPCR. (F) The correlation between MIR100HG and miR‐5590‐3p was tested by Pearson test. (G) miR‐5590‐3p expression levels after MIR100HG silencing was determined by RT‐qPCR. *p < .05. RIP, RNA immunoprecipitation; RT‐qPCR, Quantitative real‐time polymerase chain reaction.

The bioinformatics website starBase or ENCORI (https://rnasysu.com/encori/) was implemented to predict the binding site of MIR100HG to miR−5590‐3p (Figure 3A). Dual luciferase assay further examined the binding relationship between MIR100HG and miR‐5590‐3p, and the finding unveiled that the luciferase activity was significantly reduced in A549 cells cotransfected with MIR100HG‐WT and miR‐5590‐3p mimic, while cotransfection of MIR100HG‐Mut and miR‐5590‐3p mimic showed insignificant changes in cellular luciferase activity, indicating that MIR100HG could directly bind miR‐5590‐3p (Figure 3B). RIP assay demonstrated the interaction between MIR100HG and miR‐5590‐3p in A549 cells (Figure 3C).

miR‐5590‐3p expression levels in lung cancer tissues and cells were tested by RT‐qPCR, and the results signified that miR‐5590‐3p was notably downregulated in both lung cancer tissues and cells (Figure 3D,E). Pearson correlation analysis showed that MIR100HG was negatively correlated with miR‐5590‐3p (Figure 3F). Finally, RT‐qPCR was utilized to evaluate miR‐5590‐3p expression levels after MIR100HG silencing and miR‐5590‐3p expression levels were significantly upregulated after silencing MIR100HG (Figure 3G).

### 3.4 Upregulation of miR‐5590‐3p impedes proliferation and metastasis of lung cancer cells

**Figure 4:**
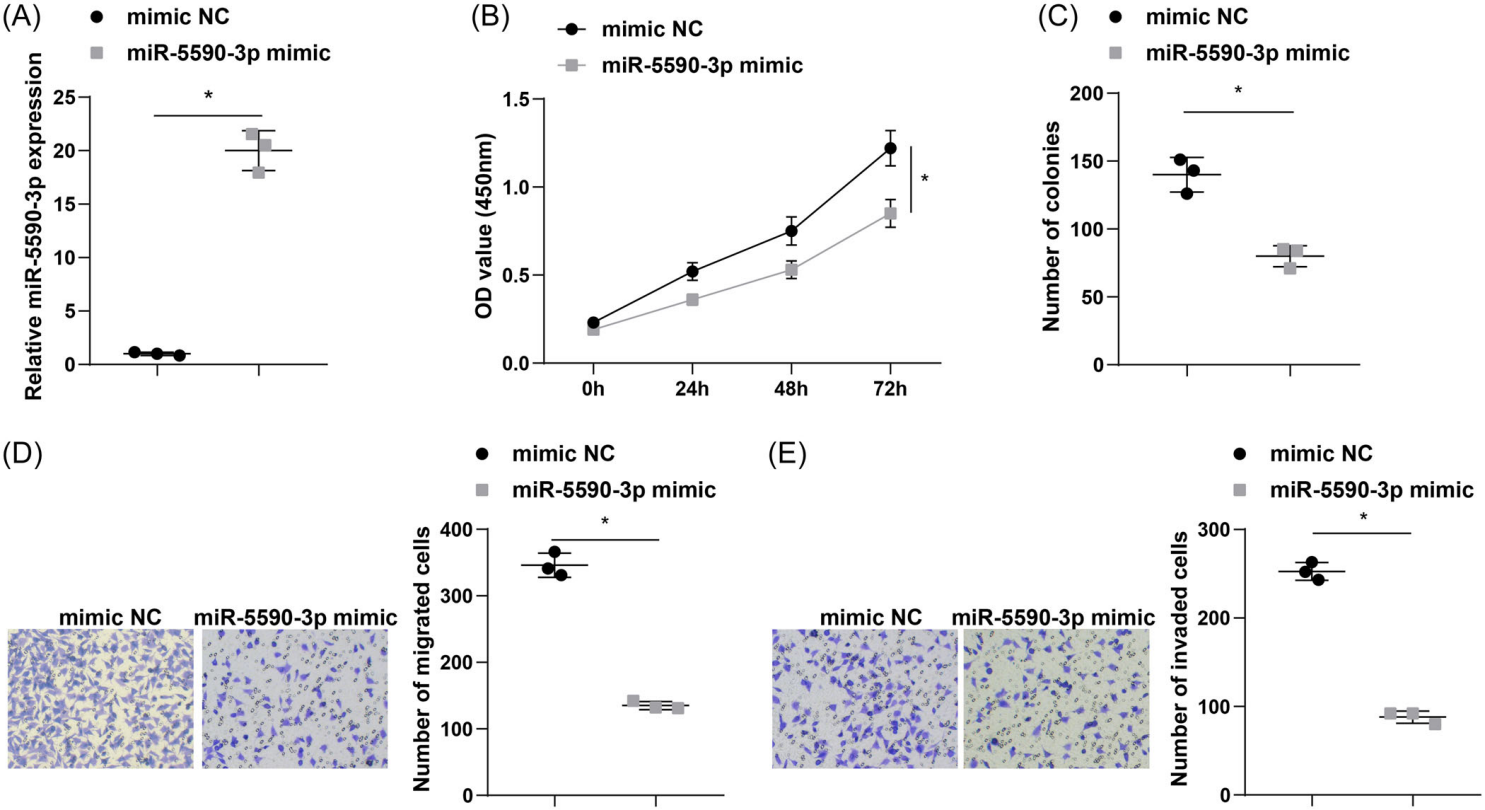
Upregulation of miR‐5590‐3p impedes proliferation and metastasis of lung cancer cells. (A) RT‐qPCR was adopted to detect miR‐5590‐3p expression levels in A549 cells. (B) CCK‐8 assay was employed to assess A549 cell proliferation ability. (C) Colony formation ability of A549 cells was examined by colony formation assay. (D and E) Transwell assay was implemented to evaluate A549 cell migration and invasion ability. *p < .05. CCK‐8, Cell Counting Kit‐8; RT‐qPCR, Quantitative real‐time polymerase chain reaction.

To investigate the influence of miR‐5590‐3p on lung cancer cell progression, miR‐5590‐3p mimic and mimic NC were transfected into A549 cells, and RT‐qPCR demonstrated successful transfection (Figure 4A). Next, we found that upregulation of miR‐5590‐3p markedly hindered cell proliferation and colony formation rate using CCK‐8 and colony formation assays (Figure 4B,C). Transwell assay revealed that upregulation of miR‐5590‐3p markedly decreased the migration and invasion properties of A549 cells (Figure 4D,E).

### 3.5 miR‐5590‐3p targets and negatively regulates DCBLD2

**Figure 5:**
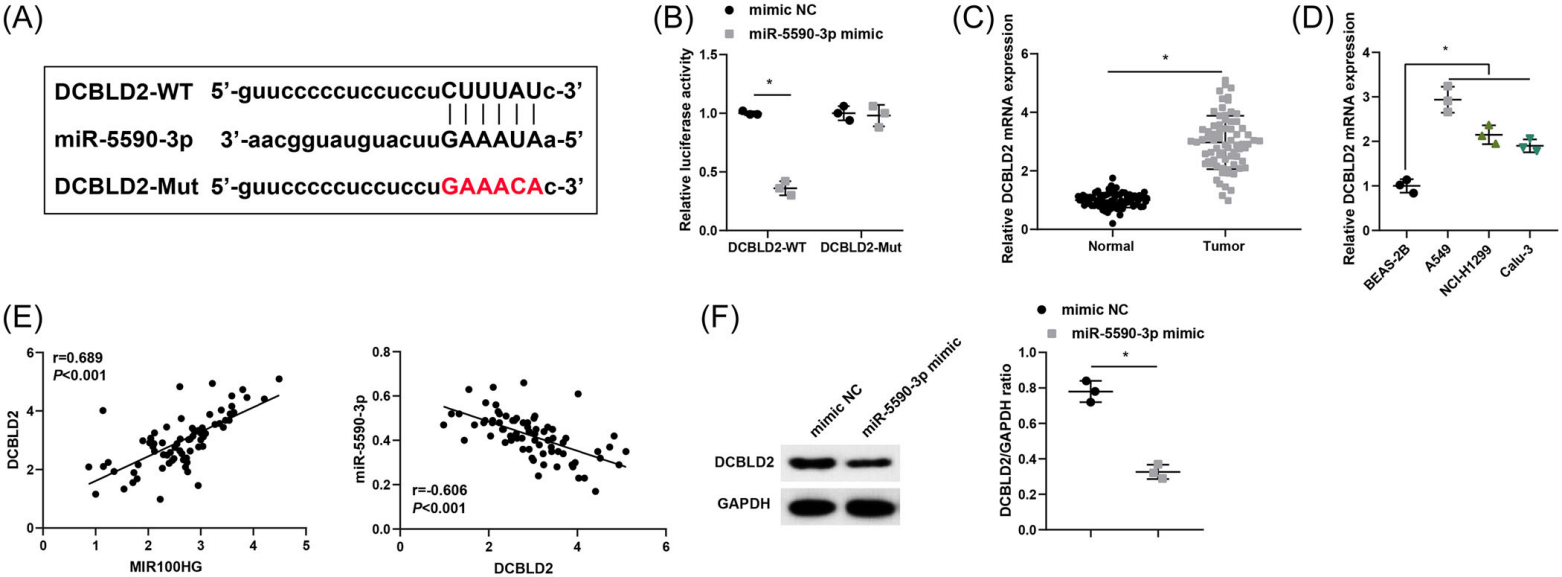
miR‐5590‐3p targets and negatively regulates DCBLD2. (A) The binding site of miR‐5590‐3p to DCBLD2 was predicted by the bioinformatics website. (B) The binding of miR‐5590‐3p to DCBLD2 was detected by a dual luciferase assay. (C) DCBLD2 expression levels in lung cancer tissues and corresponding paracancerous normal tissues were determined by RT‐qPCR (*n* = 72). (D) DCBLD2 expression levels in lung cancer cells were measured by RT‐qPCR. (E) Associations of MIR100HG and DCBLD2, as well as miR‐5590‐3p and DCBLD2, were tested by Pearson correlation analysis. (F) Protein expression levels of DCBLD2 were evaluated by western blot. *p < .05. RT‐qPCR, Quantitative real‐time polymerase chain reaction.

We further explored the downstream mechanism of miR‐5590‐3p, and DCBLD2 was predicted to be the target gene of miR‐5590‐3p using bioinformatics website starBase or ENCORI (Figure 5A). Dual luciferase assay further confirmed the binding relationship between miR‐5590‐3p and DCBLD2, and the results disclosed that luciferase activity was significantly reduced in cells cotransfected with miR‐5590‐3p mimic and DCBLD2‐WT, demonstrating that miR‐5590‐3p directly targeted DCBLD2 (Figure 5B).

DCBLD2 expression levels in lung cancer tissues and corresponding paracancerous normal tissues were examined by RT‐qPCR, and the findings revealed that DCBLD2 expression was notably upregulated in lung cancer tissues (Figure 5C). DCBLD2 was also significantly upregulated in lung cancer cells compared with BEAS‐2B cells (Figure 5D). Pearson correlation analysis suggested that MIR100HG was positively correlated with DCBLD2, and miR‐5590‐3p was negatively correlated with DCBLD2 (Figure 5E). RT‐qPCR and western blot further revealed DCBLD2 expression levels were distinctly downregulated in A549 cells after transfection with miR‐5590‐3p mimic (Figure 5F,G).

### 3.6 LncRNA MIR100HG promotes lung cancer progression by negatively regulating DCBLD2 through binding with miR‐5590‐3p

**Figure 6:**
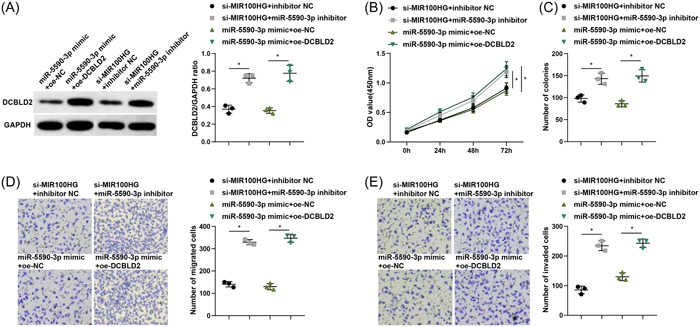
LncRNA MIR100HG promotes lung cancer progression by negatively regulating DCBLD2 through binding with miR‐5590‐3p. (A) Western blot was employed to measure the protein expression levels of DCBLD2 in A549 cells. (B) CCK‐8 assay was employed to assess A549 cell proliferation ability. (C) Colony formation ability of A549 cells was examined by colony formation assay. (D and E) Transwell assay was implemented to evaluate A549 cell migration and invasion ability. *p < .05. CCK‐8, Cell Counting Kit‐8; lncRNA, long noncoding RNA.

We further performed a rescue experiment and divided A549 cells into si‐MIR100HG + inhibitor NC group, si‐MIR100HG + miR‐5590‐3p inhibitor group, miR‐5590‐3p mimic + oe‐NC group, and miR‐5590‐3p mimic + oe‐DCBLD2 group. By RT‐qPCR and western blot assay, it was found that both DCBLD2 mRNA and protein expression levels were significantly elevated in cells of the si‐MIR100HG + miR‐5590‐3p inhibitor group and miR‐5590‐3p mimic + oe‐DCBLD2 group compared with the si‐MIR100HG + inhibitor NC group and miR‐5590‐3p mimic + oe‐NC group, respectively (Figure 6A,B). CCK‐8, colony formation, and Transwell assays were performed to estimate lung cancer cell proliferation, migration, and invasion, and the findings reflected that downregulation of miR‐5590‐3p reversed the inhibitory effect of silencing MIR100HG on proliferation, migration, and invasion of A549 cells, and overexpression of DCBLD2 also reversed the inhibitory effect of miR‐5590‐3p upregulation on cell proliferation and metastasis (Figure 6C–F).

## 4 DISCUSSION

The incidence and mortality of lung cancer are high in China, which is a major public health problem and brings a huge burden on society.^32^ Over the past two decades, our recognition of disease biology, utilization of predictive biomarkers, and therapeutic improvements have improved significantly and changed outcomes in many patients.^1^ However, the molecular mechanisms underlying lung cancer tumorigenesis and development remain to be elucidated.^33^ Therefore, in this current research, we aimed to probe the impact of MIR100HG on the proliferation and metastasis of lung cancer cells by mediating the miR‐5590‐3p/DCBLD2 axis. Collectively, we highlighted that MIR100HG promoted lung cancer progression by targeting and negatively regulating DCBLD2 through binding with miR‐5590‐3p.

The aberrant expression of lncRNAs is responsible for tumor development, particularly lung cancer.^34^ Recently, lncRNAs have been demonstrated to be related to tumour progression and the immune microenvironment.^35^ In our article, we found that MIR100HG was highly expressed in lung cancer tissues and cells, and high expression of MIR100HG was associated with late TNM stage, presence of LNM, and low differentiation in lung cancer patients. Additionally, silencing MIR100HG impeded lung cancer cell proliferation, migration, and invasion. Similar to our findings, it has been reported that MIR100HG is upregulated in idiopathic pulmonary fibrosis, and MIR100HG knockdown alleviates bleomycin‐induced lung fibrogenesis in mice and TGF‐β1‐triggered fibrotic changes in alveolar epithelial cells.^36^ Additionally, Li et al.^37^ have found that MIR100HG expression is high in colorectal cancer (CRC) tissues, and a higher MIR100HG expression is noted in advanced CRC. Besides, upregulated MIR100HG advanced CRC cell activities and liver metastatic colony formation in mice. Another paper has demonstrated that high MIR100HG expression has a positive correlation with the Edmondson‐Steiner grading and TNM stage in hepatocellular carcinoma (HCC) patients, and depletion of MIR100HG impedes the HCC cell viability, migration, as well as invasion.^38^ In the meantime, MIR100HG expression has been revealed to be positively related the tumor and clinical grades of bladder cancer patients, and restoration of MIR100HG boosts the biological functions of bladder cancer cells.^39^ Li et al.^12^ have supported that MIR100HG and ERK axis can modulate the immune system activity and thus should be regarded as chemical or antibody target for immunotherapy in the future. These findings imply that MIR100HG could therefore be thought as a prognostic hallmark and a novel therapeutic target in human diseases.

In addition, lung cancer patients were divided into high and low‐expression groups based on the median value of MIR100HG expression levels to analyze the correlation between MIR100HG levels and different clinicopathological characteristics, which demonstrated that high MIR100HG expression was associated with late TNM stage and the presence of LNM. Further Kaplan‐Meier analysis showed that lung cancer patients with low MIR100HG expression had significantly higher survival rates than those with high MIR100HG expression. Similarly, evidence has shown that elevated MIR100HG expression is associated with the Edmondson‐Steiner grading and TNM tumor stage in HCC patients.^38^ Another study has revealed that high MIR100HG expression is positively related to T stage, LNM, distant metastasis, AJCC stage, as well as histological differentiation in CRC samples.^37^ These findings imply that MIR100HG overexpression may serve a role in tumor progression and metastasis.

As previously reported, lncRNAs can sponge some miRNAs, thus alter the abundance of miRNA and hinder downstream target gene expression.^40^ To date, miRNAs regulate multipe cellular activities associated with cancer development, such as the escape from the antitumor immune response.^41^ In our research, we uncovered that MIR100HG could adsorb and bound with miR‐5590‐3p and thus inhibited its expression. In line with our finding, MIR100HG is validated to target miR‐5590‐3p, and its expression has a negative correlation with miR‐5590‐3p.^14^ Besides, we also observed that miR‐5590‐3p was lowly expressed in lung cancer tissues and cells, and elevating miR‐5590‐3p expression impeded lung cancer cell proliferation, migration, as well as invasion. Similarly, it is reported that miR‐5590‐3p is downregulated in triple‐negative breast cancer (TNBC), and elevation of miR‐5590‐3p suppresses TNBC cell proliferation and migration, as well as boosts cell apoptosis.^19^ Liu et al.^42^ have supported that miR‐5590‐3p is downregulated in renal cell carcinoma (RCC), and transfection of miR‐5590‐3p mimic hinders RCC cell proliferative, migratory, and invasive capacities. Furthermore, we also found that miR‐5590‐3p targeted and negatively regulated DCBLD2 expression. DCBLD2 has been revealed to be overexpressed in CRC tissues, and the overexpression of DCBLD2 links with a higher AJCC grade, a higher vascular invasion incidence, a poorer histological differentiation degree, as well as a poorer overall survival in CRC patients.^43^ DCBLD2 might be a potential immunological, oncogenic, as well as prognostic hallmark in terms of pan‐cancer, which could contribute to the tumor prognosis improvement and the targeted therapy development.^44^

In summary, the novelty of this study is that MIR100HG promotes lung cancer progression by modulating the miR‐5590‐3p/DCBLD2 axis. Besides, the downregulation of miR‐5590‐3p reversed the suppressive effect of silencing MIR100HG on lung cancer cell proliferation and metastasis, and the overexpression of DCBLD2 also reversed the effect of overexpression of miR‐5590‐3p on lung cancer cell proliferation and metastasis. Our work highlights that MIR100HG could therefore be thought of as a prognostic hallmark and a novel therapeutic target in human diseases.

## AUTHOR CONTRIBUTIONS

**Shengping Min**: Writing—original draft; writing—review & editing. **Linxiang Zhang**: Writing—original draft; writing—review & editing. **Li Zhang**: Writing—original draft; writing—review & editing. **Fangfang Liu**: Writing—original draft; writing—review & editing. **Miao Liu**: Data curation; writing—original draft; writing—review & editing.

## CONFLICT OF INTEREST STATEMENT

The authors declare no conflict of interest.

## ETHICS STATEMENT

All patients have signed a written informed consent form, and in addition, this study has been approved by the ethics committee of Bengbu Medical College([2023] 389).

## Data Availability

The data sets used and/or analyzed during the current study are available from the corresponding author on reasonable request.
